# Epigenetic regulation of HGK/MAP4K4 in T cells of type 2 diabetes patients

**DOI:** 10.18632/oncotarget.7686

**Published:** 2016-02-24

**Authors:** Huai-Chia Chuang, Jun-Sing Wang, I-Te Lee, Wayne H-H. Sheu, Tse-Hua Tan

**Affiliations:** ^1^ Immunology Research Center, National Health Research Institutes, Zhunan, Taiwan; ^2^ Division of Endocrinology and Metabolism, Taichung Veterans General Hospital, Taichung, Taiwan; ^3^ Faculty of Medicine, National Yang-Ming University, Taipei, Taiwan; ^4^ Department of Pathology and Immunology, Baylor College of Medicine, Houston, Texas, USA

**Keywords:** type 2 diabetes, HGK methylation, HGK downregulation, IL-6-producing T cells, Immunology and Microbiology Section, Immune response, Immunity

## Abstract

Type 2 diabetes (T2D) is a complex and heterogeneous disease. Obesity increases the risk of obese T2D; but in Asia non-obese T2D is prevalent. The cause of non-obese T2D has remained elusive. We studied the potential involvement of HGK/MAP4K4 in T2D using clinical samples from newly diagnosed, drug-naïve patients and healthy controls. HGK levels fell and IL-6 levels increased in T cells from T2D patients. Frequencies of IL-6-producing T cells were correlated with glucose levels after glucose-tolerance tests (but not body mass index and waist circumference) and inversely correlated with HGK expression levels. Moreover, methylation frequencies of the HGK promoter were increased in T2D patients and correlated with glucose levels after glucose-tolerance tests. The correlation was independent of body mass index. Demethylation treatment increased HGK expression levels and reduced IL-6 production in T2D T cells. This report identifies HGK methylation/downregulation in T cells as a potential biomarker for non-obese T2D.

## INTRODUCTION

One of the major risk factors for type 2 diabetes (T2D) is obesity. However, not all obese individuals develop diabetes, and not all T2D patients are obese (body mass index (BMI) greater than 30) [1]. According to WHO criteria for body obesity (BMI ≥ 30), non-obese T2D comprises about 80% of T2D cases in Asia, 55% of those in Europe, and 20% of those in North America and Australia [1-3]. In addition, based on WHO Expert Consultation in 2002 and epidemiological studies in Asia [4], the BMI criteria in Asia are adjusted lower (normal, overweight, and obese are BMI < 23, 23 ≤ BMI < 27.5, and BMI ≥ 27.5, respectively). Although the cut-off values for obesity are decreased, there are still many non-obese patients with T2D in Asia: about 70% of the total T2D in that continent [5, 6]. Dysregulation of the immune system plays a pivotal role in the pathogenesis of T2D [7]. Besides an increase in macrophages, an increase in CD8^+^ T cells or Th1 cells in adipose tissues contributes to T2D development in high-fat diet HFD-fed mice [8, 9]. Studying immunometabolism might help develop treatment for and prevention of T2D.

HPK1/GCK-like kinase (HGK/MAP4K4) is a member of MAP kinase kinase kinase kinases (MAP4Ks), which belong to the Ste20-like serine/threonine kinase family [10, 11]. The involvement of HGK in macrophage TNF-α signaling and adipocyte insulin resistance has been reported in studies using siRNA knockdown and overexpression systems [12, 13]. In the genotyping for tagging single nucleotide polymorphisms (SNPs) of 1769 DNA samples from the peripheral blood of prediabetic Europeans [14], two SNPs in the HGK locus were found to be associated with increased glucose levels in patients, while two other HGK SNPs were associated with reduced insulin release only in lean subjects (BMI < 25) [14]. Paradoxically, a fifth SNP and one of the latter two HGK SNPs are associated with enhanced plasma IL-6 but not with TNF-α levels [14]. Thus, the relative contribution of HGK expression to the pathophysiology of T2D may be mediated by cells other than macrophages. To date, the roles of two other MAP4Ks, HPK1 (MAP4K1) and GLK (MAP4K3), in T-cell signaling and T-cell-mediated immune responses have been demonstrated [15, 16]. Recently, we studied the *in vivo* role of HGK in immune responses by generating T-cell-specific HGK conditional knockout (T-HGK cKO) mice; we found that these mice displayed higher serum IL-6 levels and spontaneously developed insulin resistance without large weight gain [17]. Thus, we hypothesized that the loss of HGK expression in T cells and the increase of IL-6-producing T cells are associated with human T2D.

## RESULTS

### Downregulation of HGK and induction of IL-6-producing T cells in the peripheral blood of T2D patients

**Figure 1:**
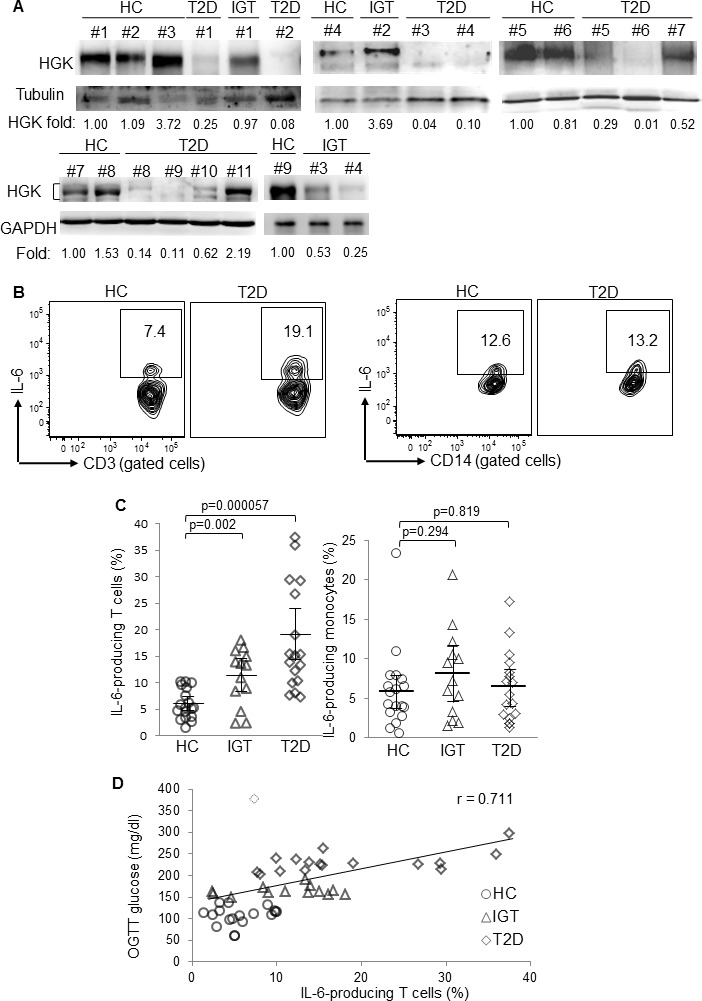
Downregulation of HGK and overproduction of IL-6 in peripheral blood T cells of T2D patients **A.** Immunoblotting analyses of indicated molecules in purified T cells from peripheral blood of HC (#1 to #9), IGT (#1 and #4), and T2D (#1 to #11). Relative fold changes of HGK were normalized to tubulin/GAPDH levels and are shown at the bottom of the figure. **B.** Flow cytometry analyses of IL-6-producing CD3^+^ T cells (left panel) and CD14^+^ monocytes (right panel) from peripheral blood of 18 healthy controls (HC), 13 impaired glucose tolerance (IGT), and 17 T2D patients; results from a T2D patient and a healthy control are shown as representative. **C.** Individual frequencies of IL-6-producing T cells (left panel) and monocytes (right panel) in three groups are shown. Bars denote mean ± 95% confidence interval. **D.** The correlation study between the frequency of IL-6-producing T cells and glucose levels after OGTT from 48 subjects. Linear regression: Y = 107.73 + 4.7992X, regression correlation coefficient: adjusted R^2^ = 0.505, r = 0.711, *P* value = 2.19 × 10^−8^, achieved power = 0.9985. The dotted diamond denotes one outlier that showed a high residual modulus value (modulus = 220.762 > 100; standardized residual = 4.173 > 3) in univariate linear regression analyses.

Because HGK deletion in T cells leads to the development of T2D in mice [17], we studied whether HGK levels are decreased in peripheral blood T cells of human T2D patients using clinical samples from drug-naïve, impaired-glucose-tolerance (IGT; a pre-diabetic state) and T2D patients, along with samples from healthy controls (Supplementary Table S1 and Supplementary Figure S1). Although intracellular staining of HGK expression using five different anti-HGK antibodies did not work with flow cytometry, immunoblotting analyses were able to detect HGK expression in T cells. Strikingly, the expression of HGK was drastically reduced in the T cells of 72% (8 in 11) of T2D patients and was modestly reduced in the T cells of some (2 in 4) IGT patients (Figure 1A).

Because an association between HGK SNPs and induced plasma IL-6 has been reported, the plasma IL-6 levels in T2D patients were determined. The plasma levels of IL-6 but not IFN-γ were significantly increased in both IGT and T2D patients (Supplementary Figure S2). To study whether IL-6 is secreted from T cells, peripheral blood leukocytes were freshly collected from 48 individuals and subjected to flow cytometry analysis (Subgroup II; Supplementary Table S1). Consistent with plasma cytokine levels, IL-6-producing T cells were significantly increased in freshly isolated peripheral blood cells of T2D patients (Figure 1B and 1C). Approximately half of IGT patients will progress to T2D over their lifetime [18, 19]. We found that the frequencies of IL-6-producing T cells were increased in 7 of total 13 IGT patients compared to healthy controls. In addition, the frequencies of IL-6-producing monocytes were not increased in IGT or T2D patients (Figure 1B and 1C). Our previous publication reported that HGK-deficient IL-6-producing T cells further differentiate into IL-6^+^ Th17 cells [17], thus, we also studied whether the IL-17 cytokine is also produced in IL-6^+^ T cells from human T2D patients. The IL-6-producing T cells from all 18 T2D patients also produced IL-17. However, the IL-6^+^ T cells from 2 of 13 IGT patients contained both IL-17^+^ and IL-17^−^ populations, while the IL-6^+^ T cell from the other 11 IGT patients were all IL-17^+^ T cells (Supplementary Figure S3). In contrast, the plasma IL-17 (or IL-6) levels were enhanced in both IGT and T2D patients compared to those in healthy controls (Supplementary Figure S2a, S2c). These results suggest that the peripheral blood IL-6-producing (IL-6^+^ IL-17^+^) T cells, instead of plasma IL-6 or IL-17 levels, are putative biomarkers for T2D.

**Figure 2:**
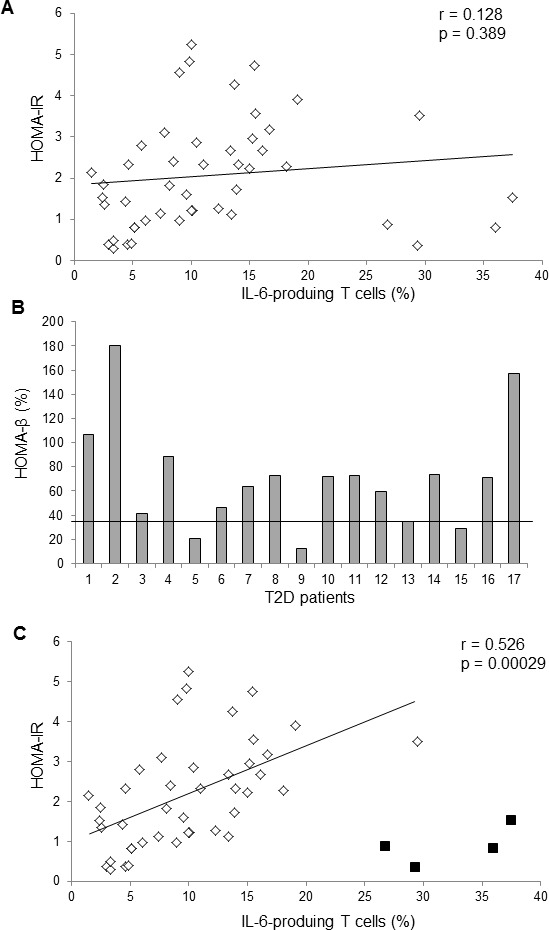
The frequencies of IL-6-producing T cells were correlated with HOMA-IR values **A.** The univariate linear regression analysis between the frequencies of IL-6-producing T cells and HOMA-IR values from 47 subjects. Linear regression: r = 0.128, *P* value = 0.398. **B.** Individual HOMA-β values of 13 T2D patients. **C.** The univariate linear regression analysis between the frequencies of IL-6-producing T cells and HOMA-IR values from 43 subjects, excluding four T2D patients' data with the lowest 25% HOMA-β values (< 35). The black squares denote four excluded T2D patients' data.

To study whether the enhancement of IL-6-producing T cells plays a role in glucose intolerance, univariate linear regression was initially used to determine the correlation between IL-6-producing T cells and OGTT glucose levels. The frequencies of IL-6-producing T cells from the 48 individuals were correlated with OGTT glucose levels (adjusted *r* = 0.587; *P* value = 1.143 × 10^−5^; unstandardized coefficient = 4.499, 95% CI, 2.659-6.3402) (Figure 1D). After removing one outlier that showed high residual value (modulus = 220.762 > 100; standardized residual = 4.173 > 3) in univariate linear regression analyses, the remaining 47 individuals showed even higher correlation (adjusted *r* = 0.711; *P* value = 2.19 × 10^−8^; unstandardized coefficient = 4.799, 95% confidence interval, 3.372-6.225) between the frequencies of IL-6-producing T cells and OGTT glucose levels (Figure 1D and Table 1). The frequencies of IL-6-producing T cells were not correlated with the values of insulin resistance index HOMA-IR (homeostatic model assessment of insulin resistance) (Table 1 lower panel and Figure 2A), perhaps because HOMA-IR calculation includes fasting insulin levels. The downregulation of fasting insulin levels in severe T2D patients suggests the development of β-cell failure. Indeed, the values of β-cell function index HOMA-β (homeostasis model assessment-β), calculated using fasting insulin levels and fasting glucose levels, were much lower in several T2D patients (Figure 2B). After excluding data of four T2D patients with the lowest 25% HOMA-β values (< 35), the frequencies of IL-6-producing T cells were in fact well correlated with HOMA-IR values (adjusted *r* = 0.526; *P* value = 0.00029; Table 1 lower panel and Figure 2C). The correlation between OGTT glucose levels and HOMA-IR values was also higher after excluding these four patients (adjusted r = 0.539; P value = 0.00019; Table 1 upper panel). Although central obesity is thought to be a risk factor for T2D in Asians [20], central obesity and BMI were not correlated with OGTT glucose levels (Table 1). Multivariate linear regression analyses further showed that individuals with IL-6-overproducing T cells were at significantly increased risk for enhancement of OGTT glucose levels after adjusting for sex, age, and BMI (standardized coefficient β = 0.641; *P* value < 0.001) or adjusting for sex, age, and waist circumference (standardized coefficient β = 0.638; P value < 0.001) (Table 2). These results suggest that IL-6-producing T cells may be involved in the pathogenesis of human T2D.

**Table 1:** Association of OGTT glucose or IL-6-producing T cells with clinical parameters (univariate linear regression analyses)

Parameters		Unstandardized coefficient (95% CI)	Linear regression coefficient (r)	*P*
OGTT glucose	IL-6-producing T cells^§^	4.799 (3.37 to 6.23)	0.711	<0.001***
	TG	−0.007 (−0.29 to 0.28)	0.010	0.957
	BMI	2.550 (−3.87 to 8.96)	0.124	0.427
	Waist	1.863 (−1.91 to 3.27)	0.083	0.600
	Fasting insulin	1.952 (−7.09 to 11.00)	0.070	0.665
	ISSI-2	−0.173 (−0.22 to −0.13)	−0.762	<0.001***
	HOMA-IR	14.761 (2.45 to 27.06)	−0.339	<0.001***
	HOMA-IR^#^	21.517 (10.92 to 32.11)	0.539	<0.001***
IL-6-producing T cells^§^	OGTT glucose	4.799 (3.37 to 6.23)	0.711	<0.001***
	TG	−0.024 (−0.06 to 0.02)	0.217	0.223
	BMI	0.188 (−0.70 to 1.07)	0.067	0.670
	Waist	0.020 (−0.33 to 0.37)	0.018	0.910
	Fasting insulin	0.013 (−0.48 to 0.51)	0.008	0.959
	ISSI-2	−0.011 (−0.01 to −0.002)	−0.357	0.023*
	HOMA-IR	0.829 (−1.09 to 2.75)	0.128	0.389
	HOMA-IR^#^	2.285 (1.12 to 3.45)	0.526	<0.001***

The clinical results — including TG, BMI, waist circumference, ISSI-2, fasting insulin, HOMA-IR, and IL-6-producing T cells — from 48 individuals (18 healthy controls, 13 IGT, and 17 T2D patients) were analyzed using univariate linear regression. §, removing one outlier that showed high residual value (modulus = 220.762 > 100; standardized residual = 4.173 > 3). #, excluding four T2D patients' data with the lowest 25% HOMA-β values (< 35). CI denotes confidence intervals. BMI, body mass index. TG, triglyceride levels. Waist, waist circumference. ISSI-2, insulin secretion-sensitivity index-2. HOMA-IR, homeostatic model assessment of insulin resistance. *, *P* value < 0.05; **, *P* value < 0.01; ***, *P* value < 0.001.

**Figure 3:**
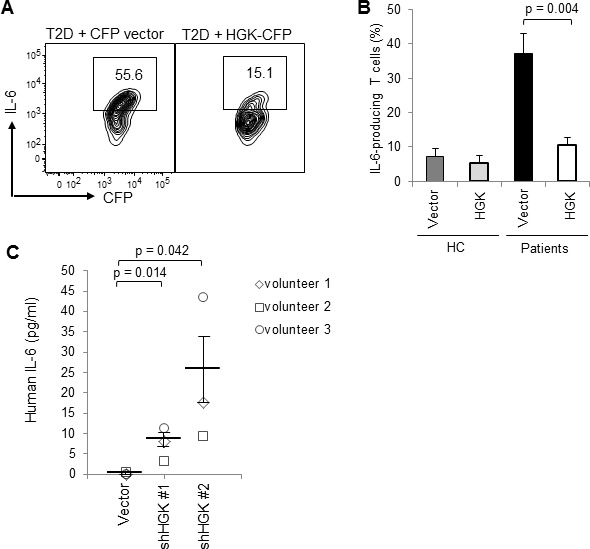
Ectopic HGK expression inhibits IL-6 production in human peripheral blood T cells **A**. T cells were isolated from the peripheral blood and transfected with a control CFP vector or HGK-CFP plasmids. T cells were stimulated with PMA plus ionomycin at day 3 after transfection, and analyzed for IL-6 by intracellular staining and subsequent flow cytometry. Contour plots depict IL-6-producing T cells (CFP-gated). Numbers indicate the percentage of cells in the quadrants. **B**. Graphs depict data from HC (*n* = 5) and patients (*n* = 5). Data show the percentages of IL-6-producing T cells (CFP-gated) and are presented as mean ± SEM. **C.** Peripheral blood T cells purified from 3 volunteers were transfected with a control vector (pSuper) or HGK shRNA plasmids (shHGK #1 or shHGK #2). The IL-6 levels in the supernatants of the transfected but unstimulated T cells were determined by ELISA. Data are presented as mean ± SEM.

T2D patients displayed lower HGK expression levels in T cells and higher frequencies of IL-6-producing T cells compared to healthy controls (Table 3). HGK expression levels were inversely correlated with IL-6-producing T cells (r = −0.593; unstandardized coefficient = −0.077, 95% confidence interval, −0.125 to −0.029; *P* value = 0.0029). In fact, all six T2D patients whose BMI was below 23 (i.e., below normal weight in Asia) showed both HGK downregulation and IL-6 overproduction (Table 3). Conversely, HGK expression was not greatly reduced in the three T2D patients (BMI = 26.6, 26.8, and 23.9) who did not have increased IL-6-producing T cells (Table 3). To confirm that IL-6 overproduction in T cells is indeed caused by HGK downregulation, HGK was ectopically expressed in patient T cells. The frequencies of IL-6-producing T cells from insulin resistant patients were significantly decreased after ectopic HGK expression (Figure 3A and 3B; *P* = 0.004). Conversely, IL-6 levels secreted from purified peripheral blood T cells were spontaneously induced by HGK shRNA knockdown (Figure 3C), while IL-17 levels were not induced (Supplementary Figure S4). These results suggest that HGK downregulation in human T cells is responsible for IL-6 induction.

**Table 2:** Multivariate linear regression analyses of OGTT glucose with IL-6-producing T cells and clinical parameters

Parameters	Unstandardized coefficient (95% CI)	Standardized coefficient (β)	*P*
IL-6-producing T cells^§^	4.329 (2.88 to 5.78)	0.641	<0.001***
BMI	0.925 (−3.09 to 4.94)	0.051	0.644
Age	0.853 (0.29 to 1.68)	0.225	0.043
Sex	13.293 (−13.38 to 43.04)	0.114	0.295
**Parameters**	**Unstandardized coefficient (95% CI)**	**Standardized coefficient (β)**	*****P*****
IL-6-producing T cells^§^	4.307 (2.85 to 5.76)	0.638	<0.001***
Waist	0.439 (−1.05 to 1.92)	0.068	0.554
Age	0.800 (−0.01 to 1.61)	0.211	0.054
Sex	13.227 (−16.22 to 42.67)	0.101	0.370

Adjusted R square = 0.525, R = 0.753

Adjusted R square = 0.523, R = 0.752

Adjusted R square = 0.525, R = 0.753

The clinical results — including TG, BMI, waist circumference, ISSI-2, fasting insulin, HOMA-IR, and IL-6-producing T cells — from 48 individuals (18 healthy controls, 13 IGT, and 17 T2D patients) were analyzed using multivariate linear regression. CI denotes confidence intervals. BMI, body mass index. Waist, waist circumference. §, removing one outlier that showed high residual value (modulus = 220.762 > 100; standardized residual = 4.173 > 3) in Table 1. *, *P* value < 0.05; **, *P* value < 0.01; ***, *P* value < 0.001.

The clinical results — including TG, BMI, waist circumference, ISSI-2, fasting insulin, HOMA-IR, and IL-6-producing T cells — from 48 individuals (18 healthy controls, 13 IGT, and 17 T2D patients) were analyzed using multivariate linear regression. CI denotes confidence intervals. BMI, body mass index. Waist, waist circumference. §, removing one outlier that showed high residual value (modulus = 220.762 > 100; standardized residual = 4.173 > 3) in Table 1. *, *P* value < 0.05; **, *P* value < 0.01; ***, *P* value < 0.001.

**Table 3:** The comparison between HGK levels in T cells and IL-6-producing T cells from IGT and T2D patients

Patient	HGK levels (fold)	IL-6-producing T (%)	BMI
T2D #1	0.25	13.4	22.9
T2D #2	0.08	15.70	30.5
T2D #3	0.04	14.00	22.1
T2D #4	0.1	13.90	23.3
T2D #5	0.29	ND	22.2
T2D #6	0.01	37.50	21.9
T2D #7	0.52	10.40	26.8
T2D #8	0.14	11.80	22.6
T2D #9	0.11	26.80	22.2
T2D #10	0.62	10.00	26.6
T2D #11	2.19	9.00	23.9
IGT #1	0.97	15.00	28.1
IGT #2	3.69	2.49	25.2
IGT #3	0.53	11.80	21.7
IGT #4	0.25	16.70	27.5
HC #1	1.00	9.80	21.8
HC #2	1.09	10.00	21.2
HC #3	3.72	3.36	25.3
HC #4	1.00	6.09	21.8
HC #5	1.00	4.88	20.5
HC #6	0.81	9.00	28.8
HC #7	1.00	7.38	20.7
HC #8	1.53	4.57	20.2
HC #9	1.00	3.31	25.3

Shown are the level of HGK expression and the frequency of IL-6-producing T cells in individual IGT or T2D patients whose immunoblot data were available. The frequency of IL-6-producing T cells for one patient (#5) was not determined (ND). Fold denotes HGK levels of individual samples relative to that of the first healthy control on each membrane (Figure 1A).

### Enhanced methylation of the HGK promoter correlates with glucose intolerance

**Figure 4:**
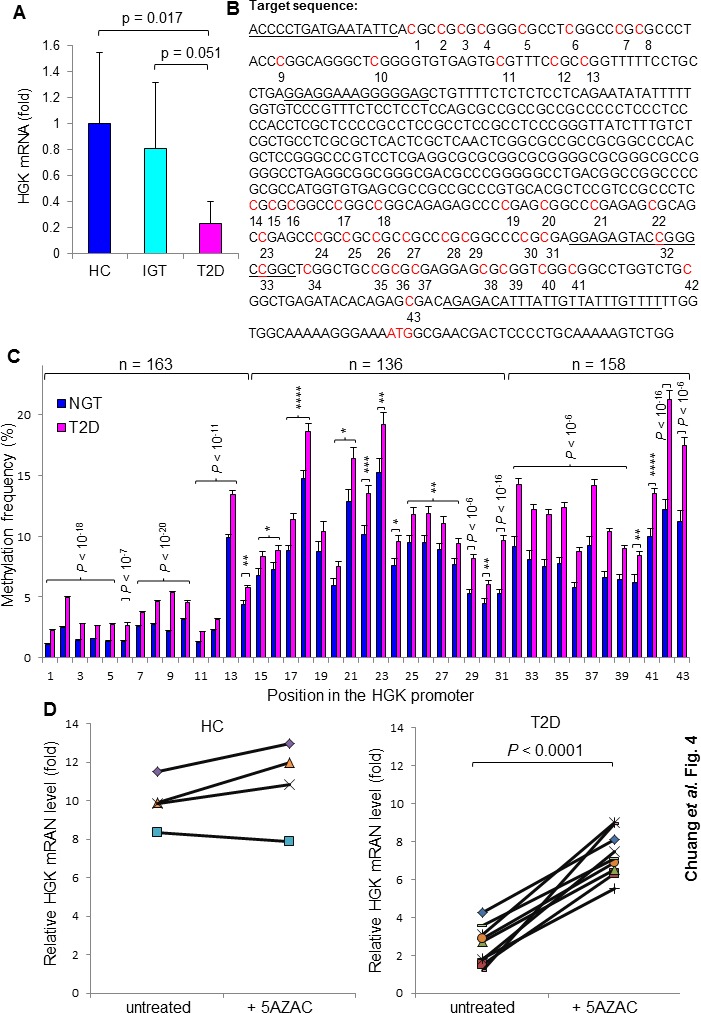
Enhanced methylation of the HGK promoter in T2D patients **A.** HGK mRNA levels in peripheral blood T cells from individuals (22 healthy controls and 21 patients) were analyzed by real-time PCR. Means ± 95% confidence intervals are shown. **B.** The position of CpG islands in the HGK promoter region. The primer location for Pyromark sequencing is underlined. **C.** Methylation frequencies of CpG sites in the HGK promoter from 163 subjects. Genomic DNA from peripheral blood mononuclear cells (PBMCs) was used. Graphs depict methylation frequencies at the positions 1-14 or positions 32-43 from NGT (*n* = 49) and T2D (*n* = 114); graphs depict methylation frequencies at the positions 15-31 from NGT (*n* = 49) and T2D (*n* = 87; data of 27 samples were undetectable). Means ± SEM are shown. **D.** Relative HGK mRNA levels in T cells from healthy controls (*n* = 4) and insulin-resistant patients (*n* = 10) before and after treatment with the methyltransferase inhibitor 5-AZAC (4 μM) for 72 hours. The HGK mRNA levels were measured by quantitative real-time PCR. *, *P* value < 0.05; **, *P* value < 0.01; ***, *P* value < 0.001; ****, *P* value < 0.0001.

Consistent with the protein levels, mRNA levels of HGK were significantly reduced in the peripheral blood T cells purified from T2D patients (Figure 4A; *P* = 0.016). We studied whether downregulation of HGK transcription is due to enhanced methylation of CpG islands in its promoter region (Figure 4B). Pyrosequencing 163 DNA samples of peripheral blood mononuclear cells (PBMCs) from normal glucose tolerance (NGT) or T2D individuals showed increased methylation frequencies at all 43 sequenced CpG sites within the HGK promoter regions in T2D patients compared to those in NGT individuals (Figure 4C). To evaluate the potential role of HGK methylation in T2D pathogenesis, we studied whether HGK methylation frequencies at individual CpG sites are correlated with any clinical parameters. We found that the methylation frequencies at many (34 of 43) positions were correlated with OGTT glucose levels (Supplementary Table S2); in particular, the correlation coefficients were high (r > 0.5) at positions 1-5 and 7-10 within the HGK promoter (Table 4). Surprisingly, the correlations between HGK methylation frequencies and OGTT glucose levels at these positions were very high (*r* = 0.603 to 0.801) in the normal (BMI ≤ 23) sub-population compared to those in the overweight or obese sub-population (Table 4). The average waist circumference of the male (*n* = 29) and female (*n* = 9) normal sub-populations (BMI ≤ 23) were 82.1 ± 6.3 cm and 74.5 ± 2.9 cm, respectively; the International Diabetes Federation's criteria for defining central obesity for Chinese men and women are waist circumference of 90 cm and 80 cm, respectively (Table 4). In contrast, HGK methylation frequencies were not correlated with age, gender, smoking status, or low-density lipoprotein levels, and were slightly correlated with BMI (at only 8 out of 43 positions; Supplementary Table S2).

**Table 4:** Association of the HGK methylation frequencies with OGTT glucose levels in three sub-populations (univariate linear regression analyses)

	All	Sub-population		
	♂Waist = 91.8 ± 8.3 cm ♀Waist = 84.8 ± 10.0 cm n = 163 (130 men)	BMI ≤ 23 ♂Waist = 82.1 ± 6.3 cm ♀Waist = 74.5 ± 2.9 cm n = 38 (29 men)	23 < BMI ≤ 27 ♂Waist = 90.8 ± 4.5 cm ♀Waist = 82.7 ± 5.5 cm n = 72 (58 men)	27 < BMI ♂Waist = 99.8 ± 5.0 cm ♀Waist = 97.0 ± 6.7 cm n = 53 (43 men)
**Pearson correlation coefficient**	OGTT glucose levels			
Pos. 1	0.562***	0.686***	0.512***	0.469*
Pos. 2	0.688***	0.801***	0.655***	0.652***
Pos. 3	0.600***	0.740***	0.553***	0.536***
Pos. 4	0.539***	0.695***	0.507***	0.448**
Pos. 5	0.564***	0.647***	0.522***	0.554***
Pos. 7	0.557***	0.658***	0.546***	0.485***
Pos. 8	0.595***	0.709***	0.546***	0.585***
Pos. 9	0.689***	0.792***	0.630***	0.709***
Pos. 10	0.513***	0.603***	0.531***	0.410**

The methylation frequencies from figure 2C and OGTT glucose levels from three sub-populations were analyzed using Pearson correlation/univariate linear regression. Pos., position. BMI, body mass index. Waist, waist circumference. *P value < 0.05; ** *P* value < 0.01; *** *P* value < 0.001.

To verify the significance of the correlation between the HGK methylation frequencies and OGTT glucose levels, the data were further analyzed using multivariate linear regression analyses. After adjusting for age, gender, BMI, and smoking status, OGTT glucose levels were indeed correlated with the methylation frequencies (at 24 in 43 positions) but not with the other 4 clinical parameters (Table 5 and Supplementary Table S3). Thus, the correlation between HGK methylation and glucose intolerance is independent of age, gender, BMI, and smoking. Taken together, our data suggest that individuals with higher methylation frequencies of the HGK promoter have high risks of glucose intolerance.

**Table 5:** Multivariate linear regression analyses for the estimated risk of high OGTT glucose levels

Parameters	Unstandardized coefficient (95% CI)		Standardized coefficient (β)	*P*
Methylation at Pos. 1	42.23	(31.90 to 52.57)	0.539	<0.001***
Age	0.23	(−0.49 to 0.96)	0.044	0.523
Sex	−18.38	(−44.80 to 8.11)	−0.105	0.173
BMI	1.26	(−1.54 to 4.05)	0.060	0.376
Smoking	−3.491	(−25.21 to 18.23)	−0.024	0.751
Methylation at Pos. 2	31.74	(26.23 to 37.25)	0.673	<0.001***
Age	0.09	(−0.55 to 0.72)	0.016	0.792
Sex	−15.49	(−38.79 to 7.81)	−0.088	0.191
BMI	0.50	(−1.97 to 2.97)	0.024	0.688
Smoking	−6.39	(−25.54 to 12.74)	−0.045	0.510
Methylation at Pos. 3	42.47	(33.03 to 51.91)	0.582	<0.001***
Age	0.35	(−0.35 to 1.05)	0.065	0.326
Sex	−12.37	(−38.19 to 13.44)	−0.071	0.345
BMI	0.83	(−1.89 to 3.56)	0.040	0.546
Smoking	−2.60	(−23.68 to 18.47)	−0.018	0.807
Methylation at Pos. 4	42.64	(31.44 to 53.84)	0.519	<0.001***
Age	0.24	(−0.50 to 0.93)	0.045	0.522
Sex	−18.17	(−45.17 to 8.84)	−0.104	0.186
BMI	0.90	(−1.97 to 3.77)	0.043	0.536
Smoking	−8.25	(−30.44 to 53.84)	−0.058	0.464
Methylation at Pos. 5	37.08	(28.06 to 46.09)	0.546	<0.001***
Age	0.06	(−0.67 to 0.78)	0.011	0.873
Sex	−21.25	(−47.59 to 5.08)	−0.121	0.113
BMI	1.00	(−1.80 to 3.80)	0.048	0.480
Smoking	−10.82	(−32.58 to 10.95)	−0.076	0.328
Methylation at Pos. 7	43.42	(32.82 to 54.01)	0.535	<0.001***
Age	0.28	(−0.45 to 1.00)	0.051	0.453
Sex	−20.89	(−47.27 to 5.48)	−0.119	0.120
BMI	1.74	(−1.04 to 4.52)	0.083	0.217
Smoking	−5.52	(−27.23 to 16.19)	−0.039	0.616
Methylation at Pos. 8	30.02	(23.40 to 36.64)	0.580	<0.001***
Age	0.25	(−0.45 to 0.95)	0.047	0.477
Sex	−22.50	(−47.99 to 3.00)	−0.128	0.083
BMI	0.99	(−1.72 to 3.70)	0.047	0.472
Smoking	−10.02	(−31.50 to 10.64)	−0.073	0.330
Methylation at Pos. 9	25.42	(21.04 to 29.79)	0.672	<0.001***
Age	0.09	(−0.55 to 0.72)	0.016	0.787
Sex	−18.28	(−41.43 to 4.88)	−0.104	0.121
BMI	0.74	(−1.71 to 3.19)	0.035	0.551
Smoking	−6.60	(−25.66 to 12.47)	−0.046	0.495
Methylation at Pos. 10	28.27	(20.61 to 35.93)	0.498	<0.001***
Age	0.41	(−0.33 to 1.16)	0.077	0.276
Sex	−25.53	(−52.60 to 1.53)	−0.146	0.064
BMI	1.47	(−1.40 to 4.34)	0.070	0.312
Smoking	−7.97	(−30.34 to 14.40)	−0.056	0.483

The methylation frequencies from Figure 2C and clinical results from all individuals (49 NGT and 114 T2D patients) were analyzed using multivariate linear regression analyses. Data are presented as standardized regression coefficient, β, and unstandardized coefficient, B (95% CI). BMI, body mass index. *, *P* value < 0.05; **, *P* value < 0.01; ***, *P* value < 0.001.

To study whether the enhanced methylation in the HGK promoter negatively regulates HGK expression, we investigated whether reversal of the epigenetic modification by demethylation restores HGK expression. The primary T cells from human subjects were treated with the methyltransferase inhibitor 5-azacytidine (5-AZAC). After demethylation treatment, the mRNA expression of HGK was increased by 3.5 fold (95% CI, 2.0 to 4.9; *P* value < 0.0001) in T cells from T2D patients (Figure 4D). In contrast, the response to 5-AZAC was abrogated in T cells from healthy controls (Figure 4D). This could be due to that the demethylation treatment cannot further reduce the already low levels of HGK methylation in T cells from healthy controls. Consistent with that, IL-6 mRNA levels were decreased in T2D T cells after demethylation treatment (Supplementary Figure S5). Demethylation of the HGK promoter in the 5-AZAC-treated T cells could not be demonstrated by pyrosquencing on the same clinical samples because only small number of peripheral blood T cells are available from drug-naïve T2D patients; therefore, other potential or indirect effects of 5-AZAC on these treated cells cannot be formally ruled out. These data suggest that enhanced methylation on the HGK promoter may cause HGK downregulation and subsequent IL-6 upregulation in T cells.

## DISCUSSION

Obesity is a major risk factor of T2D; nevertheless, not all T2D patients in Asia and Europe are obese T2D [1-3]. It appears that both obesity-dependent and obesity-independent pathways contribute to T2D. Here we propose a potential obesity-independent pathway of T2D pathogenesis. We found that HGK expression levels were decreased and IL-6 production is increased in T cells from drug-naïve, non-obese T2D patients. Notably, the frequencies of IL-6-producing T cells were correlated with OGTT glucose levels independent of either BMI or waist circumference. The methylation frequencies of the HGK promoter were correlated with OGTT glucose levels in T2D patients regardless of BMI. Moreover, the demethylation treatment restored HGK expression in T cells; ectopic HGK expression inhibited IL-6 production in patient T cells. These data are consistent with a previous report that T-cell-specific HGK knockout mice spontaneously develop non-obese T2D [17]. These findings suggest that HGK downregulation and subsequent IL-6 overproduction in human T cells may play important roles in the pathogenesis of non-obese T2D.

Recent reports have provided evidence that gastrointestinal microbiota and nutrients control the pathogenesis of T2D [21]. Moreover, environmental factors such as nutrients, hormones, and toxins regulate metabolic diseases through DNA methylation [22]. Our results showed that the correlation between HGK methylation frequencies and OGTT glucose levels in the normal sub-population was generally higher than that in the obese sub-population. Our results also showed that the correlation between HGK methylation and glucose intolerance was independent of BMI, smoking, age, and gender. These data suggest that HGK methylation and downregulation in patients enrolled in this study are not regulated by obesity, glucose, insulin, smoking, or sex hormone. However, it is possible that obesity is still a contributing factor to HGK downregulation in other ethnic T2D patients. Thus, further identification of risk factors (e.g., gastrointestinal microbiota, diet, and environmental factors) that regulate HGK methylation may help understanding of the mechanism of the T2D pathogenesis. In addition, about 25% of T2D patients enrolled in this study did not show any increase of IL-6-producing T cells or downregulation of HGK, suggesting that other pathways independent of the HGK/IL-6 pathway may also contribute to T2D in these patients.

Recent studies have highlighted an important role of T cells in regulating T2D [9, 23]. IFN-γ-producing Th1 cells were induced in HFD-fed mice [9]. Consistently, a different study found that T cells isolated from T2D patients in the United States show significantly increased IFN-γ levels after *in vitro* stimulation with PHA or anti-CD3/CD28 [23]. Furthermore, another report showed that the Th1 (T-bet^+^) to Treg ratios in adipose tissues were correlated with BMI from five patients in North America [9]. Thus, IFN-γ-producing Th1 cells may be involved in obesity-induced T2D. In contrast, the IFN-γ levels or Th1 to Treg ratios from *in vitro* stimulated patient T cells were not correlated with HbA1c levels in T2D patients in China [24]. Our results showed that drug-naïve IGT and T2D patients from Asia displayed increases in plasma IL-6 levels but not in IFN-γ levels. Importantly, the freshly isolated blood T cells from these patients showed that the frequencies of IL-6-producing T cells were highly correlated with OGTT glucose levels. Our previous publication also showed that normal diet-fed wild-type mice develop glucose intolerance after adoptive transferring with HGK-deficient IL-6-producing T cells [17]. Taken together, the numbers of IFN-γ-producing T cells and IL-6-producing T cells may reflect the severity of obese T2D and non-obese T2D, respectively. Thus, both HGK downregulation in T cells and high-fat diet are risk factors for T2D.

Non-obese T2D is prevalent in Asia [1]. Our recent study demonstrates that IL-6^+^ Th17 cells cause insulin resistance in T-cell-specific HGK conditional knockout mice without inducing obesity and that the differentiation of these pathogenic IL-6^+^ Th17 cells in adipose tissue requires a synergistic effect of IL-6 (from T cells) and leptin (from adipocytes) [17]. Moreover, IL-6 plays a critical role in the induction of high leptin levels in adipose tissue of T-cell-specific HGK conditional knockout mice [17]. Based on these findings [17], we propose that in human subjects, IL-6+ T cells could induce high levels of leptin, which in turn cooperates with IL-6 in subsequent induction of Th17 differentiation in adipose tissue microenvironment. In addition, IL-17 attenuates adipocyte accumulation by inhibiting adipogenesis [25]. Taken together, it is likely that non-obese T2D patients in Asia contain sufficient adipose tissue to drive the pathogenesis of T2D. Interestingly, several publications support this notion. Visceral, but not subcutaneous, abdominal fat is associated with insulin resistance in Indian subjects, whose average BMI is 23 in males and 24 in females [26]. Moreover, hepatic insulin resistance in USA T2D patients is correlated with visceral fat/subcutaneous fat ratio but not with BMI or total fat mass [27]. Taken together, we propose a novel hypothesis that HGK-downregulated IL-6^+^ Th17 cells promote visceral fat-mediated T2D independent of BMI. Our findings suggest that HGK/MAP4K4 in peripheral blood T cells is a useful biomarker for non-obese T2D.

## MATERIALS AND METHODS

### Study participants

A total of 228 individuals, including 73 healthy individuals and 155 patients, were enrolled in this study during 2010-2014. These 155 drug-naïve patients were newly diagnosed as having either impaired glucose tolerance (IGT) or T2D, based on the criteria of the American Diabetes Association (ADA) [19]. The glucose level after oral glucose tolerance tests (OGTT) was used as a diagnostic for IGT (140-199 mg/dl) and T2D (> 200 mg/dl). Patients with IGT were classified as prediabetic. The 67 individuals with normal glucose tolerance (NGT) and 6 healthy volunteers were used as healthy controls (HC). The main characteristics of these patients and the analysis plan of clinical samples are summarized in Supplementary Table S1 and Figure S1, respectively. The β-cell function was evaluated with the insulin secretion-sensitivity index-2 (ISSI-2) and HOMA-β. HOMA-IR, HOMA-β, and ISSI-2 were determined from the same OGTT. HOMA-IR = fasting insulin (μU/ml) × fasting glucose (mmol/l) / 22·5. HOMA-β= [20 ×fasting insulin (μU/ml)] / [fasting glucose - 3·5 (mmol/l)]. ISSI-2 = [the area under the insulin curve (AUC insulin) / the area under the glucose curve (AUC glucose)] / insulin sensitivity (Matsuda index). Matsuda index = 10,000 / [fasting glucose (mmol/l) × fasting insulin (μU/ml) × 120 min glucose (mmol/l) × 120 min insulin (μU/ml)]^0·5^ [28]. All patients were referred to the Division of Endocrinology and Metabolism at Taichung Veterans General Hospital in Taiwan. All experiments were performed in accordance with the guidelines and protocols approved by the Ethics Committee of Clinical Research, Taichung Veterans General Hospital, Taiwan. Written informed consent (approved by the Ethics Committee of Clinical Research, approval number #C08215) was obtained from all patients before enrollment in this study.

### Measurement of HGK methylation levels

Pyrosequencing was performed by Mission Biotech (Taipei, Taiwan) according to the manufacturer's instructions to validate and dissect the methylation frequencies of CpG sites in region −628 to −20 from the transcription start site of the HGK gene. The lack of pyrosequencing for some segments within this region of the HGK promoter is due to an unsuccessful primer pair for these GC-rich segments. Briefly, a biotin-labeled primer and bisulfate-converted DNA were mixed and subjected to PCR. Subsequently, the PCR products were denatured and released to single-strand products for pyrosequencing using the PyroMark Q24 system (Qiagen, Hilden, Germany). Methylation frequencies of each CpG sites were analyzed by PyroMark Q24 software.

### Reagents and plasmids

Anti-HGK antibody and HGK expression plasmids were as described previously [17]. The HGK short hairpin RNA plasmids were established by the National RNAi Core Facility (Taiwan). Anti-GAPDH and anti-tubulin antibodies were purchased from Sigma. TaqMan probe for HGK and primer sets for IL-6 were from Applied Biosystems and Bio-Rad Laboratories, respectively. Quantitative real-time PCR was performed by CFX96 Touch™ Real-Time PCR Detection System (Bio-Rad). The IL-6 and IFN-γ ELISA kits were purchased from eBioscience.

### Flow cytometry analyses

Peripheral blood leukocytes were isolated from human subjects and immediately treated with Golgi-stop, followed by cell surface staining and intracellular staining [17]. The antibodies used for staining are as follows. Anti-hCD3-PE-Cy7 (SK7), and anti-hCD3-APC-Cy7 (SK7) antibodies were purchased from BD Biosciences. Anti-hIL-6-PE (MQ-13A5) antibody was from eBioscience.

### Transient transfection of primary T cells

For transient transfection assays, primary T cells were transfected using the Neon Transfection System (Invitrogen Corp.). The settings for human primary T cells (1×10^7^) were 2200 V, duration of 20 ms, and 1 pulse.

### Statistical analyses

The normality of each column data was determined by Kolmogorov-Smirnov and Shapiro-Wilk tests using SPSS 19 software. The statistical significances between two unpaired groups were analyzed using two-tailed Student's *t*-test and two-tailed Mann-Whitney *U*-test for normally distributed data and non-normally distributed data, respectively. The relationship of two variables in the human subjects was initially studied using univariate linear regression analyses. Variables with P-values less than 0·05 on the univariate linear regression analysis were considered to be potential risk factors related to glucose intolerance. Next, multivariate linear regression analyses were used to adjust for confounders including age and sex. Power calculations were performed using G*power 3.1.6 software (available at http://www.psycho.uni-duesseldorf.de/abteilungen/aap/gpower3/download-and-register). The statistical analyses were independently verified by two biostatisticians.

## SUPPLEMENTARY INFORMATION FIGURES AND TABLES


