# Supplementary material for: Epigenetic regulation of HGK/MAP4K4 in T cells of type 2 diabetes patients
Source: Oncotarget. 2016 Feb 24;7(10):10976–89. doi: 10.18632/oncotarget.7686 (PMC4905452; doi:10.18632/oncotarget.7686)
Supplement: Supplementary file 1 [file oncotarget-07-10976-s001.pdf]

Epigenetic regulation of HGK/MAP4K4 in T cells of type 2 diabetes patients

Supplementary Information

Table of contents

Supplementary Figure 1<br>The analysis plan of clinical samples.	Page 3
Supplementary Figure 2<br>Plasma levels of IL-6 and IL-17 are significantly increased in peripheral blood of IGT and T2D patients.	Page 4
Supplementary Figure 3<br>IL-6-producing T cells in type 2 diabetes patients are also IL-17-positive T cells.	Page 5
Supplementary Figure 4<br>IL-17 levels secreted from human peripheral blood T cells are not induced by HGK shRNA.	Page 6
Supplementary Figure 5<br>Enhancement of HGK mRNA levels is concomitant with reduction of IL-6 mRNA levels in T cells after demethylation treatment.	Page 7
Supplementary Table 1<br>Clinical characteristics of overall population and the subgroup in this study.	Page 8
Supplementary Table 2<br>Association of the HGK methylation frequencies with clinical parameters.	Page 9
Supplementary Table 3<br>Multivariate linear regression analyses for the estimated risk of high OGTT glucose levels.	Page 11

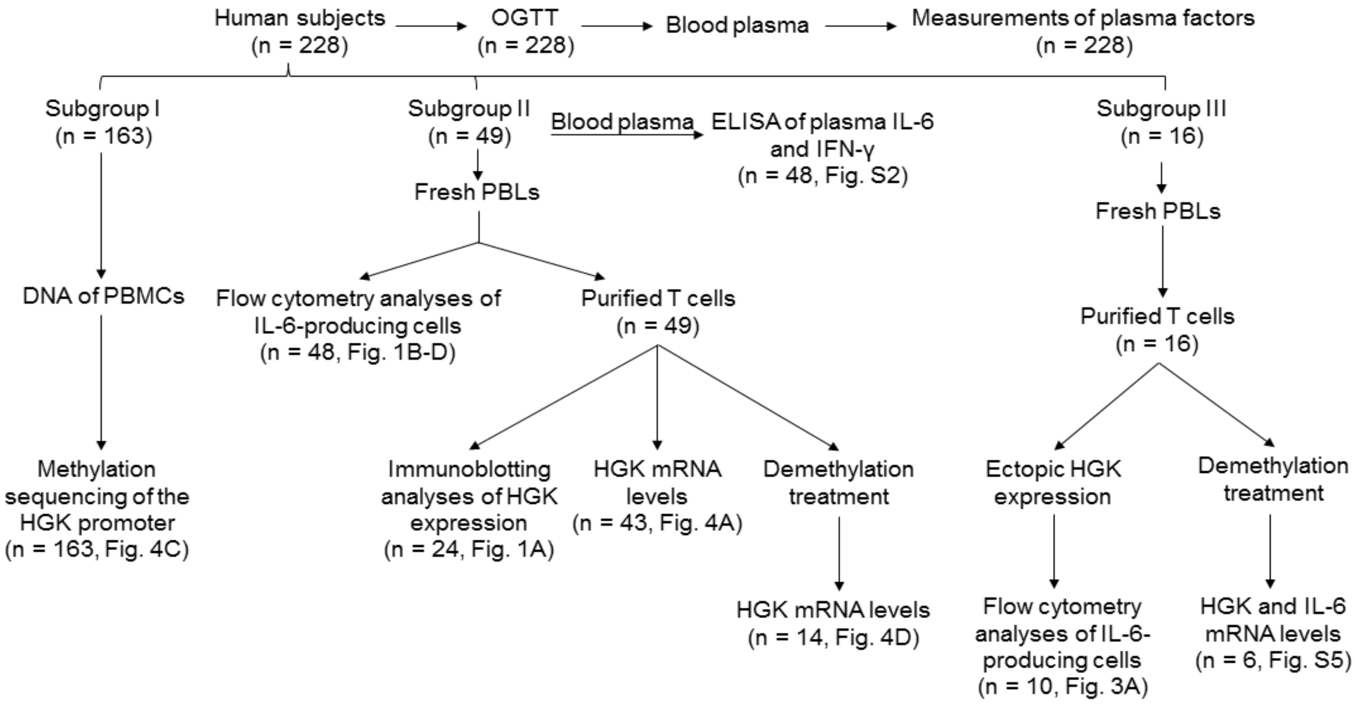

Figure S1. The analysis plan of clinical samples. OGTT, oral glucose tolerance test; PBMC, peripheral blood mononuclear cell; PBL, peripheral blood leukocytes. Plasma factors included insulin, glucose, triglyceride, glycosylated hemoglobin, and low-density lipoprotein.

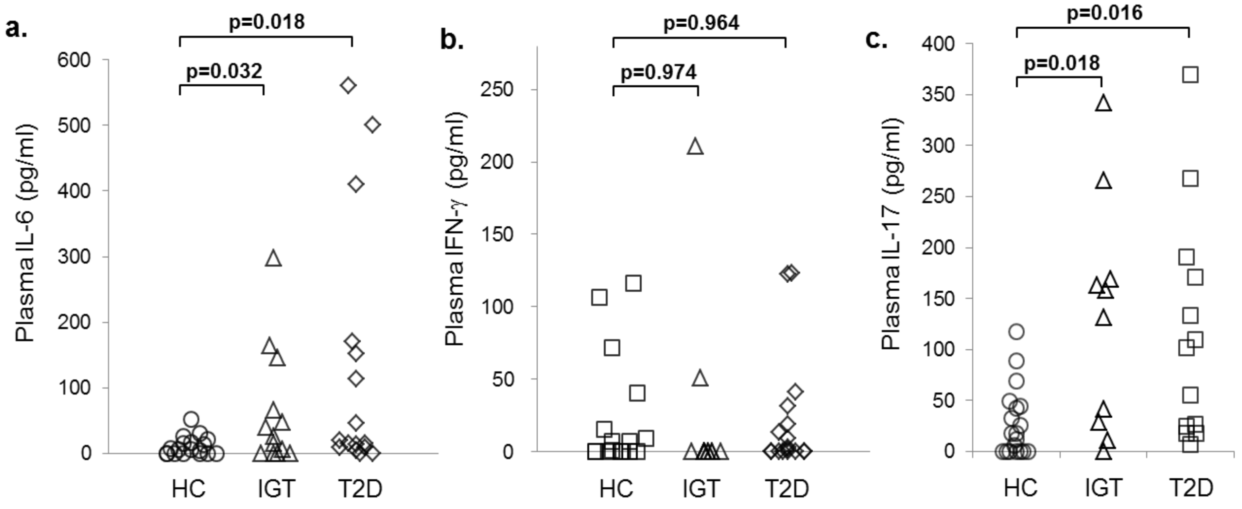

Figure S2. Plasma levels of IL-6 and IL-17 are significantly increased in peripheral blood of IGT and T2D patients. Indicated cytokine levels in the plasma of impaired glucose tolerance (IGT) patients, type 2 diabetic (T2D) patients, and healthy controls (HC) were determined by ELISA assays.

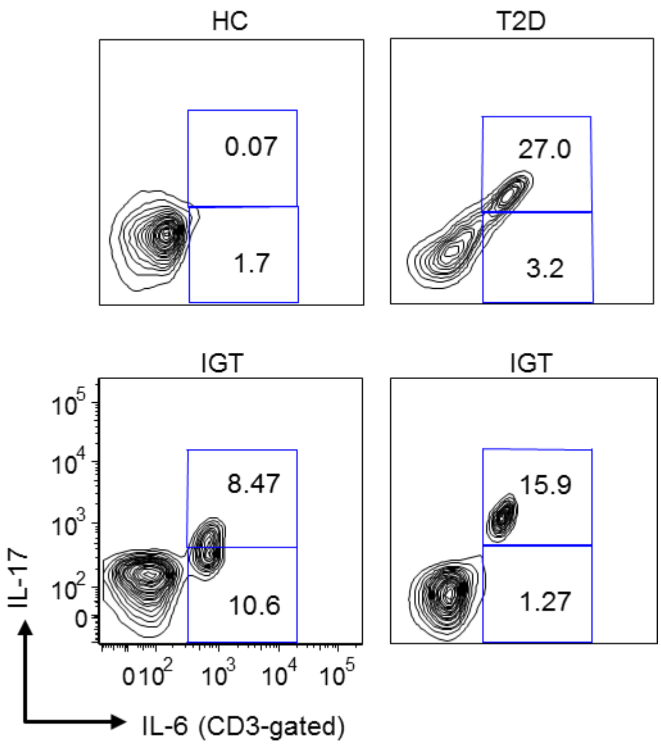

Figure S3. IL-6-producing T cells in type 2 diabetes patients are also IL-17-positive T cells. Flow cytometry analyses of IL-6-producing $CD3^+$ T cells (CD3-gated) and IL-17A-producing T cells from peripheral blood of 13 healthy controls (HC), 13 impaired glucose tolerance (IGT), and 18 type 2 diabetes (T2D) patients; representative results from one HC, two IGT, and one T2D subjects are shown.

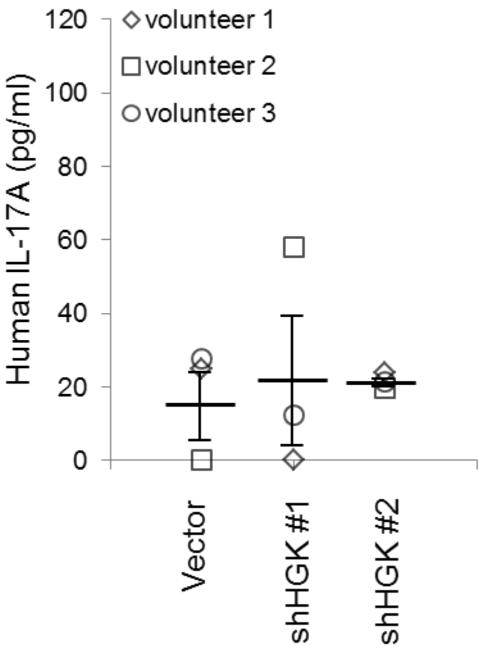

Figure S4. IL-17 levels secreted from human peripheral blood T cells are not induced by HGK shRNA. Peripheral blood T cells isolated from 3 volunteers were transfected with a control vector (pSuper) or HGK shRNA plasmids (shHGK #1 or shHGK #2). The IL-17 levels in the supernatants of the transfected but unstimulated T cells were determined by ELISA. Data are presented as mean ± SEM.

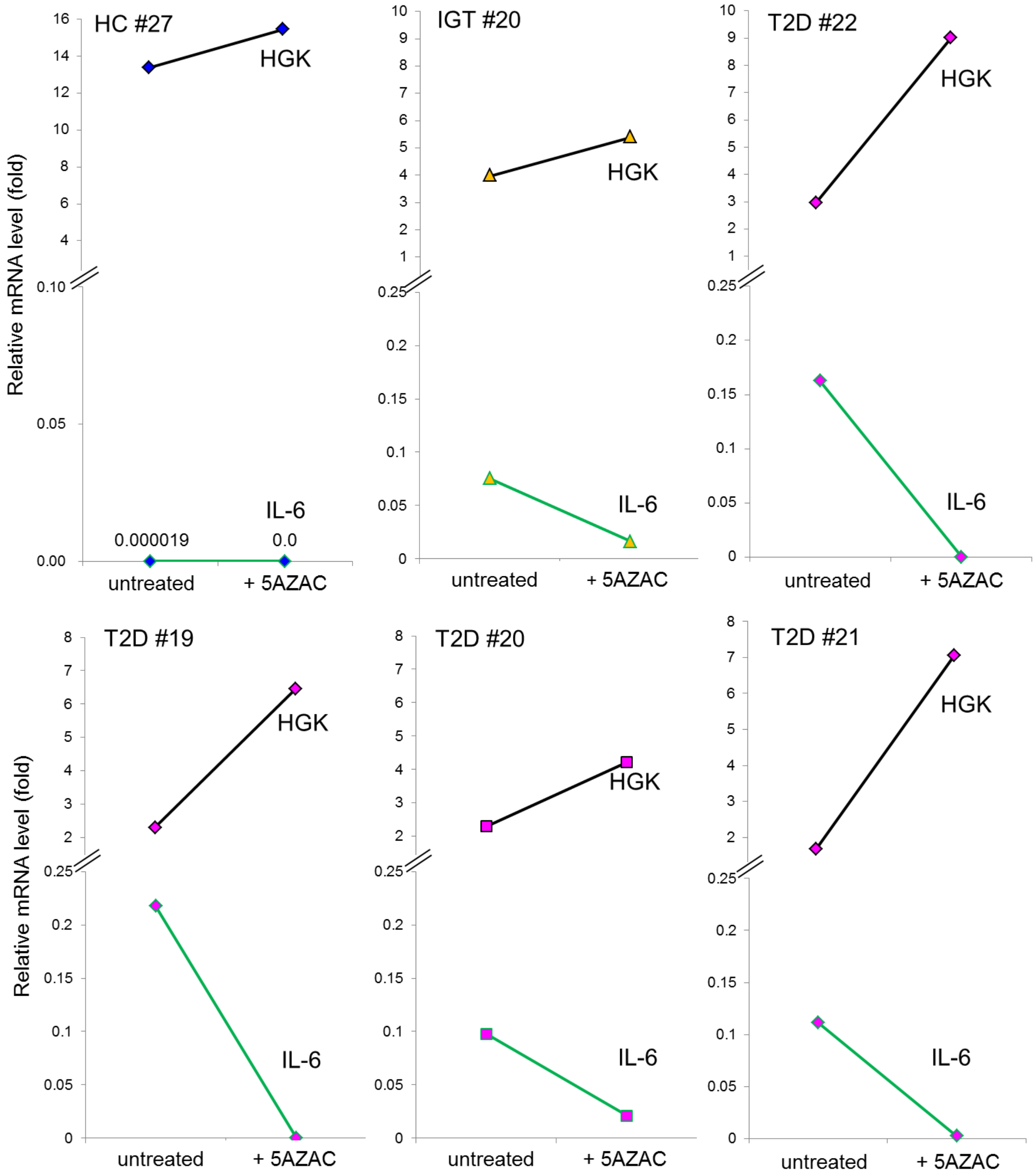

Figure S5. Enhancement of HGK mRNA levels is concomitant with reduction of IL-6 mRNA levels in T cells after demethylation treatment. Relative HGK and IL-6 mRNA levels in T cells from healthy control, IGT, and T2D patients before and after treatment with the methyltransferase inhibitor 5-azacytidine (5-AZAC; 4 µM) for 72 hours. HGK and IL-6 mRNA levels were measured by quantitative real-time PCR.

Appendix Tables

	Subgroup I		Subgroup II		
	NGT/HC	T2D	NGT/HC	IGT	T2D
**Number**	49	114	18	13	18
**Age (year)**	61.0 ± 14.5	64.2 ± 12.5	55.0 ± 9.6	59.8 ± 13.5	64.6 ± 15.2
**Sex (female/male)**	16/33	17/97	2/16	3/10	3/15
**Weight (kg)**	63.4 ± 11.4	70.1 ± 10.3	67.6 ± 10.5	69.2 ± 9.4	69.0 ± 10.3
**BMI (kg/m^2)**	24.7 ± 3.2	25.9 ± 3.4#	24.0 ± 3.1	26.6 ± 2.8*	25.1 ± 2.9
**Waist (cm)**	86.7 ± 9.3	91.9 ± 8.7	84.3 ±9 .3	91.5 ± 7.5	90.1 ± 7.8
**FPG (mg/dl)**	86.8 ± 7.5	114.7 ± 27.9#	88.9 ± 7.0	98.7 ± 6.1*	111.6 ± 14.9#
**2h PG (mg/dl)**	101.8 ± 22.3	230.7 ± 27.9#	104.6 ± 22.1	164.1 ± 10.7*	238.5 ± 41.3#
**HbA1C**	5.8 ± 0.4	6.6 ± 0.95	6.1 ± 0.4	6.2 ± 0.4	6.6 ± 0.84
**TG**	148.2 ± 87.4	162.9 ± 94.9	136.8 ± 44.3	153.8 ± 108.7	155.8 ± 80.3
**Fasting insulin (µU/ml)**	8.5 ± 6.0	10.8 ± 7.5	8.1 ± 6.3	9.9 ± 3.6	9.7 ± 5.5

Table S1. Clinical characteristics of the Subgroup I and Subgroup II in this study. Human subjects were classified based on the results of oral GTT (OGTT). Individuals with normal glucose tolerance (NGT) were used as health controls (HC). Patients with impaired glucose tolerance (IGT) were classified as prediabetic. Plus–minus values were means ± SD. *, *P* value <0.05 (comparing IGT group with HC group); #, *P* value <0.05 (comparing T2D group with HC and IGT group). BMI, body mass index. FPG, fasting plasma glucose. 2h PG, plasma glucose levels after 2h OGTT. HbA1C, glycosylated hemoglobin levels. TG, triglyceride levels. Waist, waist circumference.

Pearson correlation coefficient (P value)	Age	Sex	BMI	Smoking	LDL	OGTT Glucose	HbA1C
Pos. 1	0.028 (0.721)	-0.133 (0.091)	0.155* (0.049)	0.049 (0.534)	-0.159 (0.156)	0.562*** (0.000)	0.311*** (0.000)
Pos. 2	0.054 (0.494)	-0.149 (0.058)	0.169* (0.031)	0.071 (0.369)	-0.025 (0.749)	0.688*** (0.000)	0.290*** (0.000)
Pos. 3	-0.009 (0.913)	-0.173* (0.027)	0.184* (0.019)	0.066 (0.405)	-0.017 0.832)	0.600*** (0.000)	0.248** (0.001)
Pos. 4	0.013 (0.865)	-0.170* (0.030)	0.188* (0.016)	0.114 (0.148)	-0.059 (0.455)	0.539*** (0.000)	0.270*** (0.000)
Pos. 5	0.068 (0.390)	-0.151 (0.054)	0.159* (0.043)	0.117 (0.137)	0.016 (0.834)	0.564*** (0.000)	0.274*** (0.000)
Pos. 6	0.063 (0.422)	-0.079 (0.317)	0.007 (0.927)	0.093 (0.237)	0.120 (0.126)	0.226*** (0.004)	0.060 (0.445)
Pos. 7	0.014 (0.860)	-0.118 (0.134)	0.113 (0.152)	0.068 (0.387)	-0.019 (0.807)	0.557*** (0.000)	0.225** (0.004)
Pos. 8	0.001 (0.990)	-0.122 (0.121)	0.160* (0.041)	0.109 (0.167)	-0.006 (0.944)	0.595*** (0.000)	0.261** (0.001)
Pos. 9	0.053 (0.498)	-0.126 (0.108)	0.152 (0.053)	0.063 (0.424)	0.001 (0.986)	0.689*** (0.000)	0.292** (0.000)
Pos. 10	-0.050 (0.528)	-0.085 (0.280)	0.152 (0.053)	0.088 (0.265)	0.003 (0.973)	0.513*** (0.000)	0.242** (0.002)
Pos. 11	0.018 (0.815)	-0.162* 0.039	0.154* (0.049)	0.153 (0.051)	0.012 (0.883)	0.496*** (0.000)	0.246** (0.002)
Pos. 12	0.010 (0.895)	-0.063 (0.426)	0.122 (0.119)	0.040 (0.611)	-0.033 (0.677)	0.411*** 0.000)	0.191* (0.015)
Pos. 13	0.012 (0.879)	-0.081 (0.304)	0.131 (0.097)	0.053 (0.500)	-0.065 (0.407)	0.445*** (0.000)	0.173* (0.027)
Pos. 14	0.064 (0.462)	0.005 (0.955)	-0.003 (0.977)	-0.119 (0.166)	0.020 (0.815)	0.217* (0.011)	0.183* (0.033)
Pos. 15	-0.085 (0.325)	0.001 (0.987)	0.006 (0.941)	0.077 (0.373)	-0.042 (0.631)	0.187* (0.029)	0.275** (0.001)
Pos. 16	-0.073 (0.399)	0.036 (0.679)	0.052 (0.550)	0.040 (0.641)	0.067 (0.442)	0.181* (0.035)	0.241** (0.005)
Pos. 17	0.074 (0.392)	0.085 (0.322)	-0.034 (0.697)	-0.092 (0.288)	-0.070 (0.416)	0.238** (0.005)	0.175* (0.042)
Pos. 18	0.073 (0.401)	0.028 (0.749)	0.019 (0.828)	-0.067 (0.442)	-0.025 (0.769)	0.282** (0.001)	0.178* (0.038)
Pos. 19	0.047 (0.590)	0.090 (0.295)	-0.021 (0.806)	-0.149 (0.084)	0.112 (0.194)	0.009 (0.918)	0.176* (0.041)
Pos. 20	-0.036 (0.676)	0.037 (0.672)	-0.044 (0.609)	0.022 (0.800)	-0.001 (0.987)	0.095 (0.269)	0.226** (0.008)
Pos. 21	0.016 (0.849)	0.133 (0.123)	-0.009 (0.917)	-0.185* (0.031)	0.039 (0.649)	0.144 (0.094)	0.167 (0.052)
Pos. 22	0.059 (0.491)	-0.023 (0.786)	0.082 (0.345)	-0.079 (0.358)	0.060 (0.489)	0.202* (0.018)	0.194* (0.024)
Pos. 23	0.057 (0.513)	0.036 (0.674)	0.032 (0.715)	-0.110 (0.202)	0.085 (0.323)	0.146 (0.089)	0.189* (0.028)
Pos. 24	0.051 (0.559)	0.060 (0.486)	-0.025 (0.775)	-0.073 (0.399)	0.053 (0.537)	0.115 (0.181)	0.186* (0.030)

Pos. 25	0.084 (0.330)	0.087 (0.312)	-0.038 (0.664)	-0.155 (0.071)	0.058 (0.500)	0.137 (0.111)	0.184* (0.032)
Pos. 26	0.058 (0.505)	0.057 (0.511)	-0.042 (0.629)	-0.120 (0.164)	0.049 (0.575)	0.146 (0.089)	0.201* (0.019)
Pos. 27	0.066 (0.445)	0.101 (0.244)	-0.026 (0.767)	-0.139 (0.105)	0.061 (0.478)	0.141 (0.103)	0.184* (0.032)
Pos. 28	0.050 (0.565)	0.081 (0.349)	0.009 (0.913	-0.092 (0.284)	0.039 (0.653)	0.145 (0.093)	0.204* (0.017)
Pos. 29	0.062 (0.474)	-.071 (0.409)	0.026 (0.767)	-0.010 (0.911)	0.072 (0.402)	0.313*** (0.000)	0.295** (0.000)
Pos. 30	0.067 (0.436)	-0.016 (0.858)	-.020 (0.821)	-.053 (0.538)	0.034 (0.693)	0.196* (0.022)	0.243** (0.004)
Pos. 31	-0.003 (0.975)	-0.134 (0.120)	0.202* (0.018)	0.113 (0.191)	0.005 (0.957)	0.484*** (0.000)	0.208* (0.015)
Pos. 32	0.008 (0.918)	-0.029 (0.718)	0.015 (0.853)	-0.010 (0.903)	0.035 (0.661)	0.335*** (0.000)	0.236** (0.003)
Pos. 33	0.019 (0.813)	0.002 (0.975)	-0.005 (0.953)	-0.028 (.723)	0.014 (0.857)	0.346** (0.000)	.234** 0.003)
Pos. 34	0.016 (0.841)	-0.080 (0.315)	-0.009 (0.908)	0.020 (0.800)	0.031 (0.696)	0.427** (0.000)	0.290** (0.000)
Pos. 35	0.079 (0.323)	-0.075 (0.348)	0.045 (0.575)	-0.019 (0.815)	0.037 (0.647)	0.384*** (0.000)	0.222** (0.005)
Pos. 36	0.053 (0.507)	-0.020 (0.801)	0.038 (0.633)	-0.021 (0.792)	0.045 (0.570)	0.363*** (0.000)	0.260** (0.001)
Pos. 37	-0.041 (0.611)	-0.067 (0.401)	0.023 (0.772)	0.033 (0.679)	-0.011 (0.889)	0.408** (0.000)	0.279** (0.000)
Pos. 38	-0.030 (0.711)	-0.092 (0.247)	0.066 (0.412)	0.074 (0.352)	0.037 (0.644)	0.462*** (0.000)	0.315** (0.000)
Pos. 39	-0.027 (0.734)	-0.061 (0.447)	0.060 (0.451)	0.013 (0.866)	0.067 (0.403)	0.359*** (0.000)	0.226** (0.004)
Pos. 40	-0.080 (0.319)	-0.010 (0.904)	0.038 (0.631)	0.001 (0.993)	0.040 (0.613)	0.221** (0.005)	0.240** (0.002)
Pos. 41	-0.043 (0.591)	-0.072 (0.370)	0.031 (0.699)	0.060 (0.455)	-0.014 (0.865)	0.338** (0.000)	0.286** (0.000)
Pos. 42	0.068 (0.397)	-0.040 (0.614)	0.048 (0.548)	-0.060 (0.455	0.036 (0.651)	0.466*** (0.000)	0.252** (0.001)
Pos. 43	0.010 (0.902)	-0.126 (0.113)	0.080 (0.313)	0.019 (0.812	0.057 (0.479)	0.383*** (0.000)	0.236** (0.003)

Table S2. Association of the HGK methylation frequencies with clinical parameters (univariate linear regression analyses). The methylation frequencies from figure 4C and clinical results from all individuals (49 NGT and 114 T2D patients) were analyzed using Pearson correlation/univariate linear regression. BMI, body mass index. LDL, low-density lipoprotein. HbA1C, glycosylated hemoglobin levels. Waist, waist circumference. *, P value < 0.05; **, P value < 0.01; ***, P value < 0.001.

Parameters	Unstandardized coefficient (95% CI)	Standardized coefficient (β)	P
Methylation at Pos. 6	6.99 (2.03 to 11.95)	0.213	0.006**
Age	0.26 (-0.58 to 1.11)	0.049	0.538
Sex	-29.23 (-59.77 to 1.31)	-0.167	0.061
BMI	2.97 (-0.24 to 6.17)	0.141	0.069
Smoking	-5.81 (-31.11 to 19.49)	-0.041	0.651
Methylation at Pos. 11	51.36 (36.32 to 66.37)	0.477	<0.001***
Age	0.22 (-0.54 to -0.98)	0.040	0.574
Sex	-22.22 (-49.85 to 5.42)	-0.127	0.114
BMI	1.36 (-1.57 to 4.29)	0.065	0.361
Smoking	-11.68 (-34.57 to 11.21)	-0.082	0.315
Methylation at Pos. 12	26.69 (16.97 to 36.41)	0.391	5.425
Age	0.29 (-0.50 to 1.08)	0.054	0.470
Sex	-27.19 (-55.91 to 1.54)	-0.155	0.063
BMI	2.02 (-1.02 to 5.06)	0.096	0.191
Smoking	-4.80 (-28.52 to 18.92)	-0.34	0.70
Methylation at Pos. 13	8.01 (5.35 to 10.66)	0.423	<0.001***
Age	0.28 (-0.50 to 1.06)	0.052	0480
Sex	-25.86 (-54.15 to 2.43)	-0.148	0.073
BMI	1.85 (-1.14 to 4.85)	0.88	0.223
Smoking	-5.39 (-28.74 to 17.97)	-0.038	0.649
Methylation at Pos. 14	-0.65 (-4.50 to 3.19)	-0.026	0.737
Age	0.35 (-0.51 to 1.21)	0.065	0.422
Sex	-30.15 (-61.42 to 1.12)	-1.72	0.59
BMI	3.08 (-2.20 to 6.36)	0.147	0.65
Smoking	-3.41 (-29.38 to 22.56)	-0.024	0.796
Methylation at Pos. 15	-0.12 (-2.48 to 2.24)	-0.008	0.919
Age	0.35 (-0.51 to 1.21)	0.065	0.426
Sex	-30.11 (-61.44 to 1.23)	-0.172	0.060
BMI	3.08 (-0.20 to 6.36)	0.147	1.855
Smoking	-2.90 (-28.75 to 22.94)	-0.20	0.825
Methylation at Pos. 16	-0.51 (-2.87 to 1.86)	-0.033	0.674
Age	0.34 (-0.52 to 1.20)	0.063	0.437
Sex	-29.66 (-61.03 to 1.70)	-0.169	0.064
BMI	3.11 (-0.18 to 6.39)	0.148	0.063
Smoking	-2.86 (-28.68 to 22.97)	-0.20	0.827
Methylation at Pos. 17	-0.09 (-2.08 to 1.91)	-0.007	0.933
Age	0.35 (-0.51 to 1.21)	0.66	0.419
Sex	-30.09 (-61.49 to 1.30)	-0.172	0.060
BMI	3.08 (-0.20 to 6.36)	0.147	0.066
Smoking	-3.00 (-28.87 to 22.87)	-0.021	0.819
Methylation at Pos. 18	-0.13 (-1.43 to 1.17)	0.015	0.847
Age	0.35 (-0.51 to 1.21)	0.066	0.420
Sex	-30.04 (-61.36 to 1.28)	-0.171	0.060
BMI	3.09 (-0.19 to 6.37)	0.147	0.065
Smoking	-3.07 (-28.93 to 22.79)	-0.021	0.815
Methylation at Pos. 19	-0.98 (-2.45 to 0.49)	-0.103	0.189
Age	0.35 (0.51 to 1.20)	0.065	0.422
Sex	-29.06 (-60.21 to 2.10)	-0.166	0.067
BMI	3.04 (-0.22 to 6.31)	0.145	0.068
Smoking	-4.80 (-30.64 to 21.05)	-0.034	0.714
Methylation at Pos. 20	-0.79 (-3.19 to 1.60)	-0.051	0.514
Age	0.34 (-0.52 to 1.20)	0.063	0.440
Sex	-29.46 (-60.78 to 1.86)	-0.168	0.065
BMI	3.05 (-2.3 to 6.33)	0.145	0.068
Smoking	-2.89 (-28.69 to 22.90)	-0.20	0.825
Methylation at Pos. 21	-0.174 (-1.35 to 1.01)	-0.023	0.771

Age	0.35 (-0.51 to 1.21)	0.65	0.426
Sex	-29.84 (-61.21 to 1.52)	-0.170	0.062
BMI	3.08 (-0.21 to 6.36)	0.146	0.066
Smoking	-3.41 (-29.43 to 22.61)	-0.024	0.796
Methylation at Pos. 22	-0.28 (-1.83 to 1.27)	-0.028	0.718
Age	0.352 (-0.51 to 1.21)	0.66	0.420
Sex	-30.19 (-61.46 to 1.072)	-0.172	0.058
BMI	3.12 (-0.17 to 6.40)	0.148	-0.063
Smoking	-3.33 (-29.24 to 22.58)	-0.023	0.800
Methylation at Pos. 23	-0.36 (-1.42 to 0.70)	-0.053	0.502
Age	0.35 (-0.51 to 1.21)	0.065	0.420
Sex	-29.82 (-61.07 to 1.43)	-0.170	0.061
BMI	3.11 (-0.17 to 6.39)	0.148	0.063
Smoking	-3.70 (-29.60 to 22.19)	-0.26	0.778
Methylation at Pos. 24	-0.79 (-2.82 to 1.25)	-0.060	0.447
Age	0.35 (-0.51 to 1.21)	0.066	0.420
Sex	-29.39 (-60.68 to 1.90)	-0.168	0.065
BMI	3.07 (-0.21 to 6.34)	0.146	0.66
Smoking	-3.45 (-29.27 to 22.37)	-0.024	0.792
Methylation at Pos. 25	-0.75 (-2.58 to 1.08)	-0.064	0.420
Age	0.35 (-0.51 to 1.21)	0.066	0.417
Sex	-29.40 (-60.67 to 1.88)	-0.168	0.065
BMI	3.05 (-0.22 to 6.33)	0.145	0.068
Smoking	-4.02 (-29.93 to 21.89)	-0.028	0.760
Methylation at Pos. 26	-0.67 (-2.47 to 1.14)	-0.057	0.468
Age	0.35 (-0.51 to 1.21)	0.065	.425
Sex	-29.65 (-60.91 to 1.61)	-0.169	0.063
BMI	3.05 (-0.23 to 6.33)	0.145	0.068
Smoking	-3.81 (-29.70 to 22.09)	-0.027	0.772
Methylation at Pos. 27	-0.64 (-2.54 to 1.26)	-0.052	0.507
Age	0.35 (-0.51 to 1.21)	0.066	0.419
Sex	-29.39 (-60.71 to .1.94)	-0.168	0.066
BMI	3.07 (-0.21 to 6.34)	0.146	0.066
Smoking	-3.70 (-29.59 to 22.20)	-0.026	0.778
Methylation at Pos.28	-1.20 (-3.59 to 1.21)	-0.077	0.328
Age	0.35 (-0.51 to 1.21)	0.065	0.423
Sex	-28.94 (-60.23 to 2.34)	-0.165	0.070
BMI	3.10 (-0.17 to 6.37)	0.148	0.063
Smoking	-3.65 (-29.45 to 22.14)	-0.026	0.780
Methylation at Pos. 29	0.83 (-1.86 to 3.51)	0.047	0.544
Age	0.35 (-0.51 to 1.21)	0.066	0.419
Sex	-30.12 (-61.36 to 1.13)	-0.172	0.059
BMI	3.06 (-0.22 to 6.34)	0.146	0.067
Smoking	-2.56 (-28.39 to 23.28)	-0.018	0.845
Methylation at Pos.30	0.10 (-3.00 to 3.20)	0.005	0.950
Age	0.35 (-0.51 to 1.21)	0.066	0.420
Sex	-30.22 (-61.50 to 1.06)	-0.172	0.058
BMI	3.08 (-0.20 to 6.36)	0.147	0.065
Smoking	-2.89 (-28.78 to 23.00)	-0.020	0.826
Methylation at Pos.31	2.29 (-0.11 to 4.68)	0.147	0.061
Age	0.36 (-0.49 to 1.21)	0.068	0.400
Sex	-29.88 (-60.81 to 1.05)	-0.171	0.058
BMI	2.63 (-0.65 to 5.91)	0.125	0.116
Smoking	-3.66 (-29.21 to 21.90)	-0.026	0.778
Methylation at Pos.32	3.48 (1.77 to 5.18)	0.298	<0.001***
Age	0.40 (-0.42 to 1.22)	0.074	0.341
Sex	-29.06 (-58.84 to 0.72)	-0.166	0.056
BMI	2.86 (-0.27 to 5.98)	0.136	0.073
Smoking	-1.33 (-25.93 to 23.28)	-0.009	0.915

Methylation at Pos.33	4.27 (2.30 to 6.23)	0.316	<0.001***
Age	0.38 (-0.43 to 1.20)	0.071	0.355
Sex	-30.58 (-60.17 to -0.99)	-0.175	0.043*
BMI	2.96 (-0.15 to 6.06)	0.141	0.062
Smoking	-1.08 (-25.53 to 23.38)	-0.008	0.931
Methylation at Pos.34	5.41 (3.39 to 7.43)	0.379	<0.001***
Age	0.41 (-0.39 to 1.20)	0.076	0.311
Sex	-25.68 (-54-54 to 3.18)	-0.147	0.081
BMI	2.97 (-0.05 to 6.00)	0.142	0.054
Smoking	-1.12 (-24.92 to 22.69)	-0.008	0.93
Methylation at Pos.35	4.54 (2.54 to 6.54)	0.330	<0.001***
Age	0.29 (-0.52 to 1.10)	0.054	0.485
Sex	-26.29 (-55.79 to 3.20)	-0.150	0.080
BMI	2.58 (-0.52 to 5.68)	0.123	0.102
Smoking	-0.30 (-24.64 to 24.05)	-0.002	0.981
Methylation at Pos.36	6.15 (3.36 to 8.93)	0.321	<0.001***
Age	0.32 (-0.50 to 1.13)	0.059	0.443
Sex	-29.88 (-59.42 to -0.34)	-0.171	0.047*
BMI	2.64 (-0.47 to 5.75)	0.126	0.095
Smoking	-1.50 (-25.91 to 22.91)	-0.010	0.904
Methylation at Pos.37	4.37 (2.65 to 6.08)	0.364	<0.001***
Age	0.50 (-0.30 to 1.30)	0.093	0.222
Sex	-26.82 (-55.88 to 2.23)	-0.153	0.070
BMI	2.79 (-0.26 to 5.83)	0.133	0.073
Smoking	-1.92 (-25.90 to 22.06)	-0.013	0.875
Methylation at Pos.38	7.37 (4.77 to 9.96)	0.401	<0.001***
Age	0.47 (-0.32 to 1.25)	0.087	0.243
Sex	-26.74 (-55.31 to 1.84)	-0.153	0.066
BMI	2.35 (-0.66 to 5.35)	0.112	0.125
Smoking	-4.33 (-27.91 to 19.26)	-0.030	0.718
Methylation at Pos.39	6.50 (3.39 to 9.60)	0.307	4.133
Age	0.45 (-0.37 to 1.27)	0.085	0.275
Sex	-27.66 (-57.39 to 2.07)	-0.158	0.068
BMI	2.58 (-0.54 to 5.71)	0.123	0.105
Smoking	-1.48 (-26.03 to 23.06)	-0.010	0.905
Methylation at Pos.40	3.58 (0.90 to 6.26)	0.202	0.009**
Age	0.47 (-0.38 to 1.31)	0.087	0.279
Sex	-29.84 (-60.45 to 0.77)	-0.170	0.056
BMI	2.89 (-0.33 to 6.10)	0.138	0.078
Smoking	-2.02 (-27.31 to 23.27)	-0.014	0.875
Methylation at Pos.41	4.00 (1.98 to 6.02)	0.292	<0.001***
Age	0.47 (-0.36 to 1.29)	0.087	0.267
Sex	-28.26 (-58.13 to 1.61)	-0.161	0.064
BMI	2.76 (-0.37 to 5.90)	0.132	0.084
Smoking	-3.38 (-28.04 to 21.28)	-0.024	0.787
Methylation at Pos.42	3.30 (2.19 to 4.41)	0.415	<0.001***
Age	0.30 (-0.48 to 1.08)	0.056	0.448
Sex	-26.36 (-54.72 to 2.00)	-0.150	0.068
BMI	2.47 (-0.51 to 5.45)	0.118	0.104
Smoking	2.35 (-21.12 to 25.82)	0.016	0.844
Methylation at Pos.43	3.04 (1.68 to 4.41)	0.326	<0.001***
Age	0.40 (-0.41 to 1.21)	0.075	0.330
Sex	-23.21 (-52.89 to 6.47)	-0.132	0.124
BMI	2.43 (-0.68 to 5.54)	0.116	0.125
Smoking	-0.41 (-24.81 to 23.99)	-0.003	0.973

Table S3. Multivariate linear regression analyses for the estimated risk of high OGTT glucose levels. The methylation frequencies and clinical results from 163

individuals (49 NGT and 114 T2D patients) were analyzed using multivariate linear regression analyses. Data are presented as standardized regression coefficient, β, and unstandardized coefficient, B (95% CI). BMI, body mass index. *, *P* value < 0.05; **, *P* value < 0.01; ***, *P* value < 0.001.
